# A novel *ANK1* frameshift mutation associated with neonatal hereditary spherocytosis: a case report

**DOI:** 10.3389/fped.2025.1666585

**Published:** 2025-09-18

**Authors:** Xin Qing, Jimo Zhu, Xiaoshi Zhu, Yu Zhang, Junchao Deng, Binzhi Tang

**Affiliations:** ^1^ Department of Pediatrics, Sichuan Provincial People’s Hospital, School of Medicine, University of Electronic Science and Technology of China, Chengdu, China; ^2^ Department of Pediatrics, School of Medicine and Life Science of Chengdu University of Traditional Chinese Medicine, Chengdu, China

**Keywords:** ANK1 gene, frameshift mutation, hereditary spherocytosis, neonates, WES—whole-exome sequencing

## Abstract

**Background:**

Hereditary spherocytosis (HS) is a genetically inherited hemolytic anemia resulting from erythrocyte membrane defects, predominantly associated with genetic mutations in membrane protein genes such as *ANK1* and *SPTB*. The disease exhibits considerable heterogeneity in both clinical manifestations and age of onset, presenting substantial diagnostic challenges for clinicians, particularly in pediatric cases.

**Case presentation:**

The patient was a 29-day-old boy who had experienced persistent anemia and a medical history of neonatal hyperbilirubinemia since birth. Upon admission, the infant lacked typical HS manifestations such as splenomegaly, jaundice, and spherocytosis on the peripheral blood smear. Whole-exome sequencing identified a novel frameshift mutation c.3556delG (EX30, NM_000037.4), resulting in an amino acid alteration p.Glu1186Lysfs*3. Subsequent Sanger sequencing-based family segregation analysis confirmed that this mutation originated from the paternal allele. Based on the characteristic clinical manifestations and genetic findings, a definitive diagnosis of HS was established.

**Conclusions:**

In neonates presenting with unexplained recurrent anemia, particularly those with a history of neonatal hyperbilirubinemia, HS should be suspected. Due to the atypical manifestations, genetic analysis serves as a pivotal tool in the early diagnosis of HS, and novel genetic mutations may be identified, which can subsequently be added to the genetic database.

## Background

Hereditary spherocytosis (HS) is a genetically inherited hemolytic anemia caused by defects in erythrocyte membrane proteins. The clinical manifestations exhibit considerable heterogeneity in terms of age of onset, phenotypic severity, genetic mutation profiles, and inheritance patterns, thereby posing significant challenges for the early identification of HS (1). The molecular pathogenesis is primarily attributed to mutations in five genes (*ANK1*, *SPTB*, *SLC4A1*, *EPB42*, and *SPTA1*, with *ANK1* and *SPTB* being the most prevalent), which disrupt the vertical linkages of the erythrocyte membrane and induce structural abnormalities in the cytoskeletal architecture. These pathological changes lead to membrane instability, spheroidal transformation of erythrocytes, and subsequent splenic sequestration, ultimately establishing a chronic hemolytic process (2).

Epidemiological data has revealed significant geographical variation in the incidence of HS. Northern European and North American populations demonstrated higher prevalence rates, ranging from 1/2,000 to 1/5,000, whereas Asian populations exhibited a relatively lower incidence, estimated at approximately 1/100,000. However, the true incidence might be higher when considering potential underdiagnosis (3). Typical clinical manifestations include chronic hemolytic anemia, splenomegaly, and intermittent jaundice. However, pediatric manifestations exhibit greater variability, including isolated reticulocytosis, neonatal hyperbilirubinemia, recurrent jaundice or hemolytic episodes, and anemia (4). Notably, approximately 20% of sporadic cases were devoid of a definitive family history, particularly among individuals with novel mutations (5). Given the diagnostic constraints in primary healthcare settings, which arise from both technical limitations and insufficient clinical awareness, these factors contribute to persistently high rates of underdiagnosis or misdiagnosis. We present herein a neonatal HS case with a frameshift *ANK1* gene mutation that has not been previously reported, with the aim of improving the early diagnosis and management of neonatal HS, especially in patients with atypical clinical symptoms.

## Case presentation

A 29-day-old boy from Sichuan province of China was referred to pediatric outpatient department due to decreased hemoglobin (HGB) level of 72 g/L. He had been hospitalized during the first week of life due to neonatal hyperbilirubinemia and his HGB level was monitored at 89 g/L, prompting the initiation of oral iron supplementation. And following the administration of phototherapy, his jaundice subsided. However, one week later, the HGB level decreased to 61 g/L, necessitating a red blood cell (RBC) transfusion and his HGB level recovered to 109 g/L following the transfusion. Owing to this episode, the infant was subjected to additional evaluations, including thalassemia gene analysis, serum UAST (unusual antibody screening test), and neonatal hemolytic disease testing, all of which showed no abnormalities. The parents denied consanguinity, family history of genetic disorders, hematologic diseases, infectious diseases, jaundice, or hepatic disorders.

**Figure 1:**
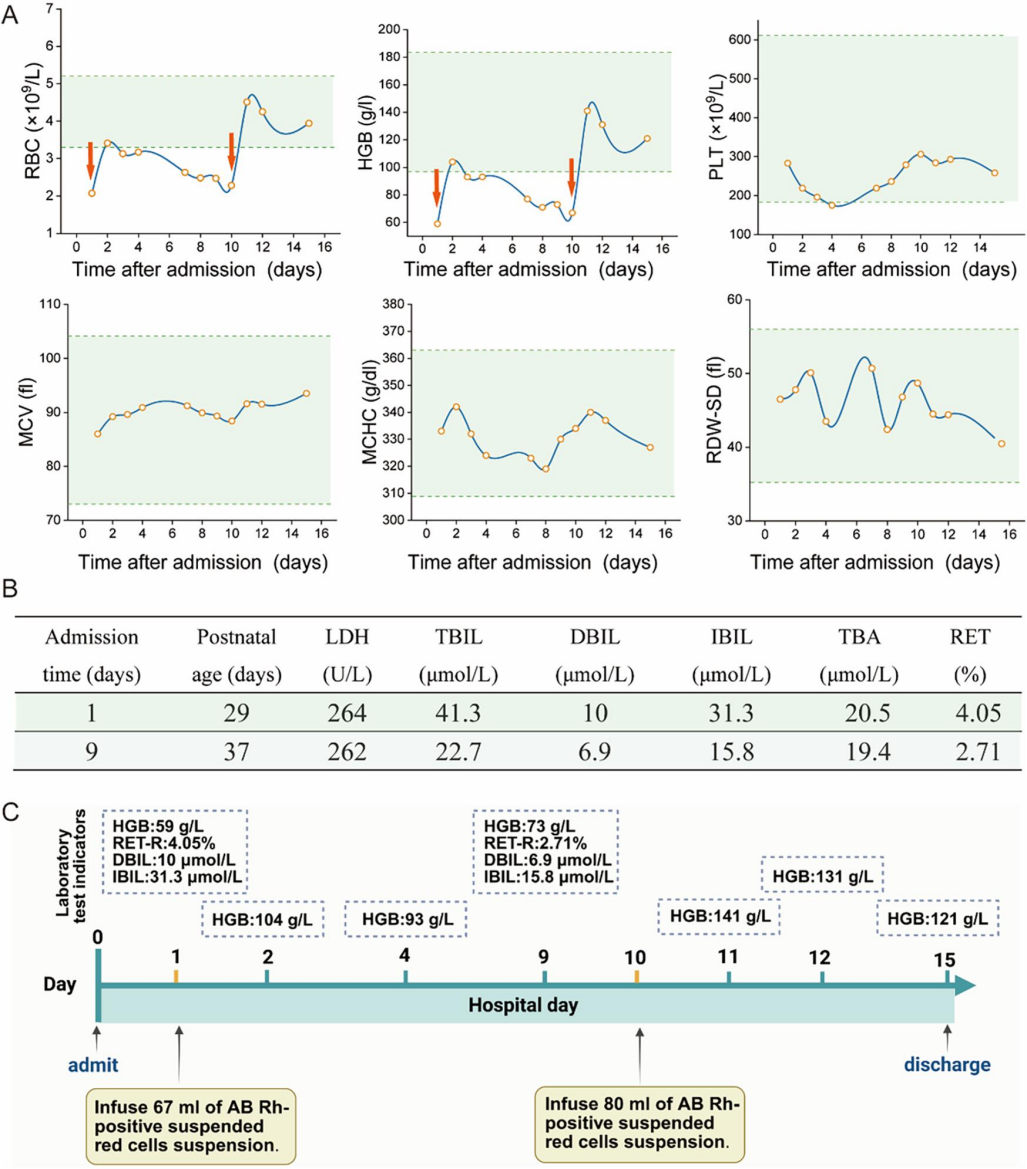
Partial auxiliary examinations for the child patient. **(A)** The main results of CBC after admission. (RBC, Red Blood Cell; HGB, Hemoglobin; PLT, Platelet; MCV, Mean Corpuscular Volume; MCHC, Mean Corpuscular Hemoglobin Concentration; RDW-SD, Red Cell Distribution Width—Standard Deviation. The red arrows represent the RBC transfusion). **(B)** The main results of liver function index and reticulocyte count (LDH, lactate dehydrogenase; TBIL, total bilirubin; DBIL, direct bilirubin; IBIL, indirect bilirubin; TBA, total serum bile acids; RET, reticulocyte count). **(C)** The laboratory test results before and after transfusion (HGB, hemoglobin; RET-R, reticulocyte count ratio; DBIL, direct bilirubin; IBIL, indirect bilirubin).

The infant exhibited no clinical signs of jaundice or hepatosplenomegaly upon admission, but mild pallor and tachycardia were noted as the only positive findings. The results of key parameters from the complete blood count (CBC) after admission were shown in Figure 1A Serum bilirubin levels, lactate dehydrogenase (LDH), total bile acids, and reticulocyte counts were summarized in Figure 1B. Additional investigations, including CD55/CD59 assays for screening of paroxysmal nocturnal hemoglobinuria, Coombs test, glucose-6-phosphate dehydrogenase (G6PD) activity assessment, urine hemosiderin analysis, vitamin B12 levels and folate concentration, were all within normal limits. Iron-metabolic related test revealed normal levels of serum iron and transferrin saturation, an elevated serum ferritin level at 610.02 ng/ml (normal reference: 21.81–274.66 ng/ml), decreased unsaturated iron-binding capacity at 18.8 µmol/L (normal reference: 20.0–62.0 µmol/L), and total iron-binding capacity at 42.8 µmol/L (normal reference: 50–77 µmol/L) respectively. Peripheral blood smear examination demonstrated hypochromic erythrocytes with enlarged central pallor. Although no spherocytes were observed, it is important to note that this finding may be limited by the constraints of automated scanning techniques. Bone marrow cytology and staining did not reveal any significant abnormalities.

**Figure 2:**
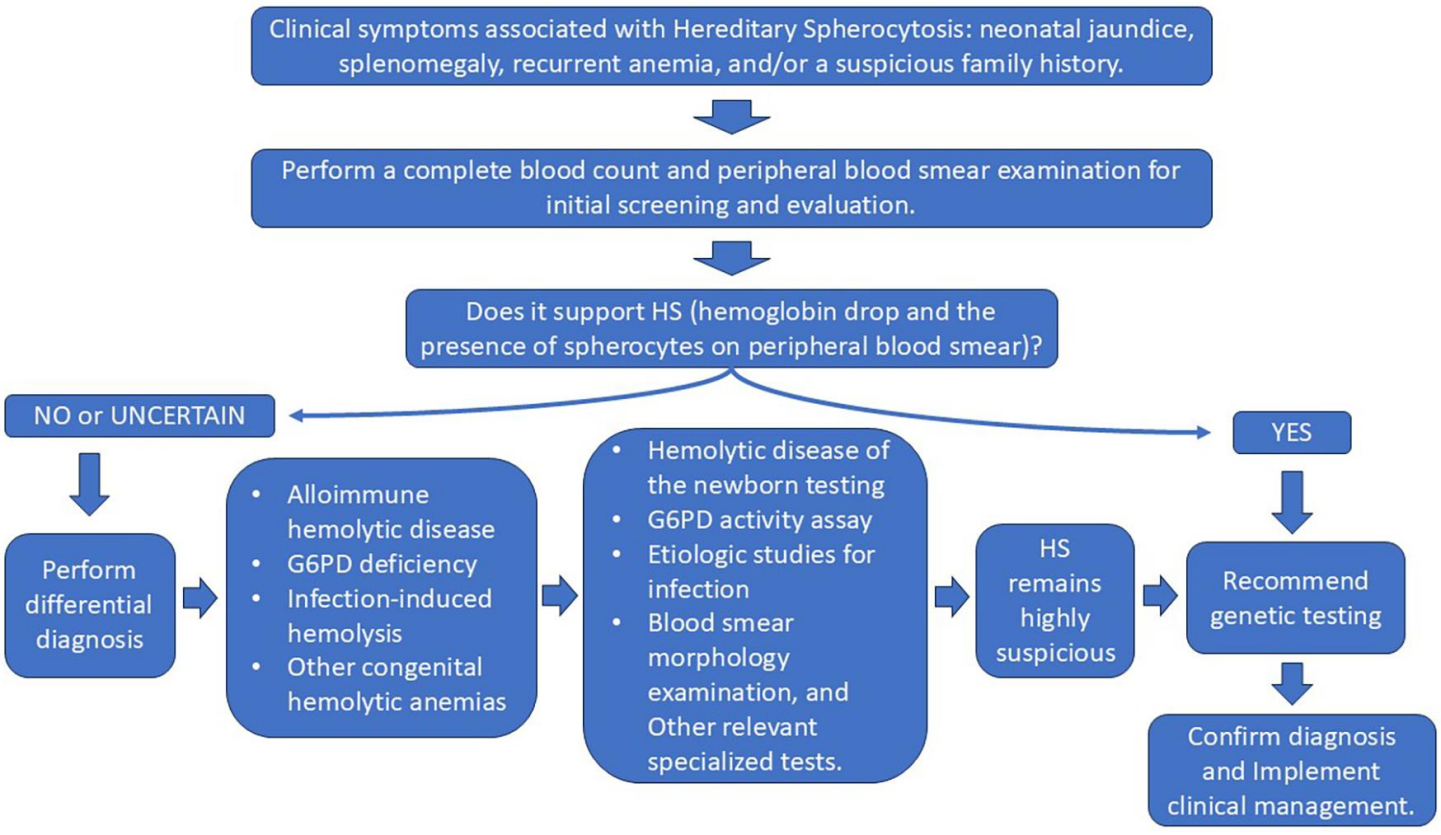
Differential diagnosis protocol for hereditary spherocytosis in newborns.

**Figure 3:**
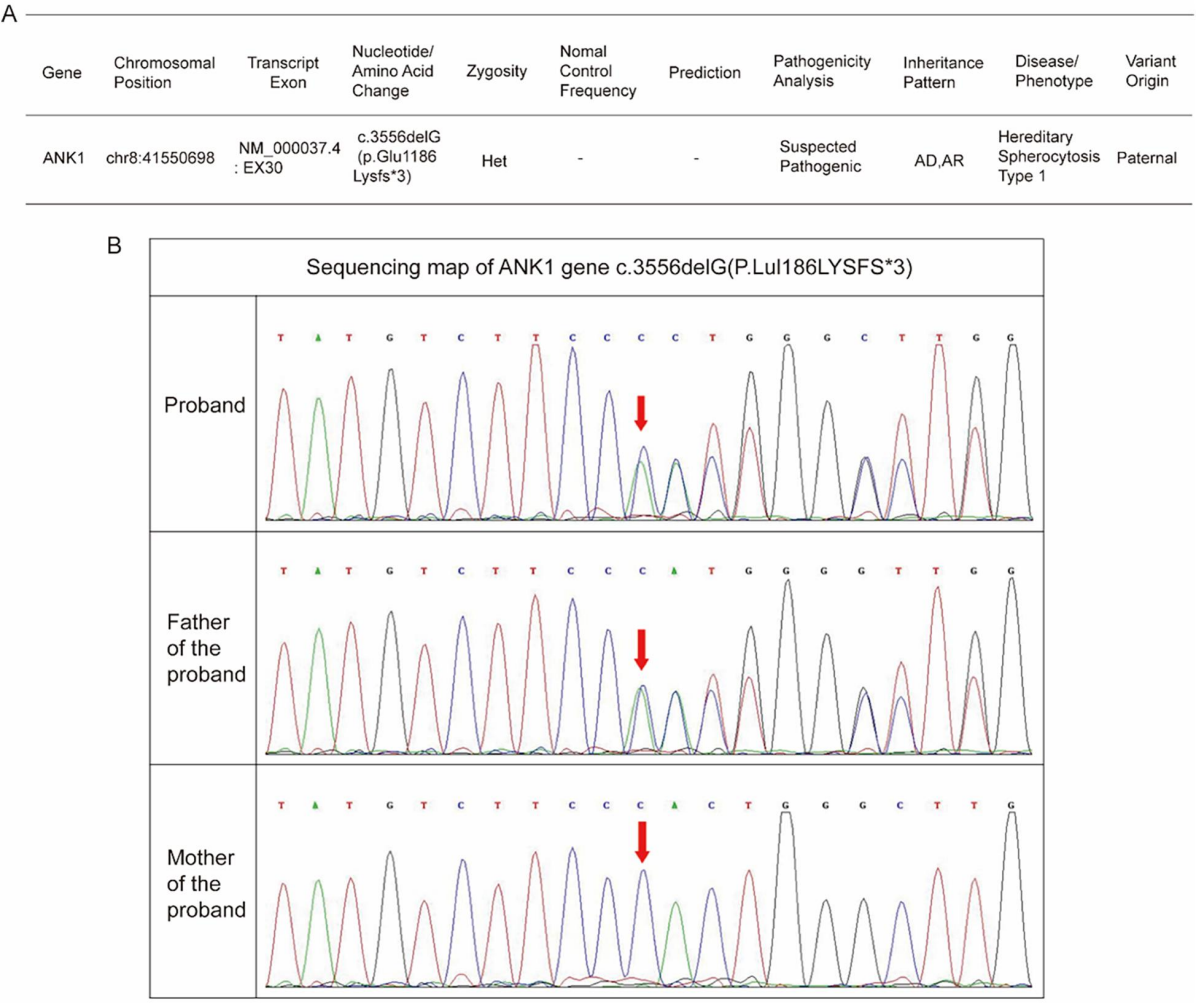
Genetic analysis of the infant. **(A)** The Whole Exome Sequencing (WES) result. **(B)** The Sanger family segregation analysis result.

The infant received RBC transfusions upon admission, which resulted in a transient improvement in anemia. However, this was followed by a progressive decline in hemoglobin levels. As illustrated in Figure 1, a second RBC transfusion was administrated 10 days after admission. Following the transfusion, hemoglobin levels stabilized, and he was subsequently discharged. Laboratory test results before and after transfusion are demonstrated in Figure 1C. Furthermore, the general workflow for performing differential diagnosis after hospital admission is illustrated in Figure 2. To investigate the etiology of the recurrent anemia, we recommended whole-exome sequencing (WES) to the family. We thoroughly addressed their concerns regarding the necessity, cost, and required blood volume for the test. The family, who shared our urgency in identifying the underlying cause of the child's condition, ultimately conveyed their understanding and granted consent to proceed with the diagnostic testing. WES identified a heterozygous frameshift variant in the *ANK1* gene: c.3556delG (p.Glu1186Lysfs*3) located at chr8:41550698 (Figure 3A), the suspected pathogenic variant is inherited in an autosomal dominant or autosomal recessive manner and can lead to HS type I. Sanger sequencing confirmed the maternal wild-type genotype and the paternal origin of the variant (Figure 3B).

After discharge, the patient was enrolled in a structured program of regular telephone follow-ups. We conduct follow-up assessments every three to four weeks and recommend monitoring through CBC tests at intervals of one to two months or whenever suspicious anemia symptoms arise. One month post-discharge, a progressive decline in hemoglobin levels was noted, necessitating a readmission to a local hospital where symptomatic treatment was provided through the administration of RBC transfusion. Following this intervention, the anemia was relieved, and he is currently undergoing routine monitoring of CBC as part of ongoing care. Considerations for splenectomy and other long-term interventions are based on the post-discharge monitoring of hemoglobin levels.

## Discussion

As a genetic disorder, HS predominantly follows an autosomal dominant inheritance pattern, while a minority exhibit autosomal recessive inheritance. Due to its highly variable clinical spectrum ranging from significant anemia to asymptomatic presentations, it poses challenges for early detection. Therefore, pre-pregnancy or prenatal screening using Whole Exome Sequencing (WES) is warranted, particularly in cases with a family history of parental anemia. Literature indicates that *ANK1* is the most prevalent pathogenic gene associated with HS, accounting for 30%–50% of cases, and represents the primary etiological factor for neonatal HS in China (6). Common mutation types encompass nonsense mutations, frameshift mutations, and splice-site mutations. Compared to other HS-related genes, *ANK1* exhibits a higher likelihood of *de novo* mutations (7). The nkyrin-1 protein, which is encoded by this gene and represents a crucial member of the ankyrin family, is located on human chromosome 8p11.2 and predominantly expressed in erythrocyte membranes. This protein functions to anchor the band 3 protein to the spectrin-actin cytoskeleton, thereby forming a stable complex that confers erythrocyte flexibility and deformability (8). Structurally, Ankyrin-1 consists of three distinct domains: an N-terminal domain containing multiple ankyrin repeats; a highly conserved central spectrin-binding domain; and a C-terminal regulatory domain prone to variation. *ANK1* mutations impair the connectivity of membrane protein, compromise the stability of the lipid bilayer, induce cytoskeletal abnormalities, and facilitate the spheroidal transformation of erythrocytes. Consequently, these alterations reduce cellular deformability, enhance splenic sequestration, and exacerbate hemolysis (9).

In the present case, the identified *ANK1* mutation lead to a frameshift at codon 1,186, leading to the generation of a premature termination codon and truncation of the C-terminal regulatory domain. Consequently, this impaired the stability of membrane skeleton formation, thereby resulting in HS-related manifestations. According to the relevant guidelines of American College of Medical Genetics and Genomics (ACMG) guidelines, the pathogenicity of the mutation site is rated as follows: (1) PVS1: This mutation is a frameshift mutation, which can cause severe abnormalities in protein structure and function; (2) PM2: This variation has not been reported in the literature and is not included in the normal population database, being a rare variation. In conclusion, this variation is a suspected pathogenic variation (PVS1 + PM2) (10). Based on the clinical manifestations, medical history, laboratory tests and genetic testing results of the child, it was diagnosed as hereditary spherocytosis type I (OMIM: 182900). Sanger sequencing-based family validation confirmed that the mutation was inherited from the father, who exhibited no clinical abnormalities or hematologic deviations on CBC analysis. This highlights the variable expressivity of HS, even among family members harboring identical mutations. This finding underscores the importance of implementing family-based genetic screening and counseling following the identification of a pathogenic variant in a proband. These measures not only facilitate the detection of asymptomatic or atypical carriers of hereditary syndromes, but also support timely clinical surveillance, minimize the likelihood of misdiagnosis, and offer critical insights for reproductive planning and long-term disease management. Notably, the child did not exhibit typical HS features such as splenomegaly or jaundice, and peripheral blood smears showed no spherocytosis, thereby complicating the diagnosis. However, a history of neonatal hyperbilirubinemia, normal results of Coombs test and G6PD assay, and the presence of severe anemia raised suspicion for atypical HS, which was subsequently confirmed by genetic analysis.

Of notice, a paucity of spherocytes or other atypical findings is frequently observed in peripheral blood smears of infants with HS. For instance, a recent study by Xie et al. reported a case of an infant with severe anemia whose peripheral smear demonstrated only polychromatophilic cells and schistocytes, necessitating regular transfusions prior to the definitive diagnosis of *ANK1*-related HS (11). Similarly, a neonate with an ANK1 missense mutation and a family history of anemia was described by Asten et al., yet only 0.8% spherocytes were revealed in peripheral blood smears (12). Additionally, it was noted by Kang et al. that among 17 cases of ANK1-related hereditary spherocytosis (HS), both jaundice and splenomegaly were absent in two cases, including a 1-month-old infant (13). These atypical clinical manifestations significantly elevate the likelihood of misdiagnosis or delayed diagnosis in infants. It is precisely the atypical clinical presentations of this disorder that underscore the importance of including it in newborn screening programs, as such inclusion could significantly reduce diagnostic delays. However, considering that the incidence of HS in China is considerably lower than that observed in Caucasian populations in Western countries, population-wide neonatal screening may lack sufficient cost-effectiveness. Nevertheless, for newborns with a family history of anemia, confirmed HS, or asymptomatic HS carriers, targeted screening using advanced genetic sequencing technologies is strongly warranted. Such an approach should be complemented by comprehensive diagnostic evaluations, including osmotic fragility testing and eosin-5-maleimide (EMA)-binding assays, to ensure accurate diagnosis.

Additionally, during the process of history-taking, in addition to family history, particular attention should be directed towards identifying whether the infant experienced hyperbilirubinemia after birth. Research has demonstrated that jaundice is the most prevalent manifestation of HS during the neonatal period, with bilirubin levels frequently surpassing the thresholds for phototherapy (14). Therefore, neonates presenting with hyperbilirubinemia, after ruling out isoimmune hemolytic disease of the newborn, should prompt consideration of suspicious HS. And continuous monitoring of hemoglobin levels is crucial to prevent complications arising from acute-onset hemolytic anemia.

Current guidelines recommend the Eosin-5′-maleimide (EMA) binding assay for HS screening (5). This method quantifies the reduction in fluorescence via flow cytometry, which results from diminished levels of the band 3 protein. However, certain limitations are evident when applying this assay to pediatric populations (15). Ciepiela et al. emphasized that the use of adult-derived reference MCV values may skew EMA results for children. Therefore, it is crucial to ensure that reference samples align with the patient's MCV within ±2 fL (16). Although the MCHC/MCV ratio or MCHC has been proposed as a supportive marker, particularly in neonates exhibiting jaundice, its clinical utility remains controversial due to insufficient validation in large-scale studies (17, 18). Recent advances in genetic sequencing have taken on a pivotal role in the diagnosis of HS, particularly in cases with positive family histories or neonatal hyperbilirubinemia, as exemplified by the present case.

For pediatric patients with moderate-to-severe HS, splenectomy remains the primary intervention for alleviating hemolysis. Current clinical guidelines generally recommend delaying splenectomy until after the age of 5, consistent with recent data from the United States indicating a growing trend toward conservative management strategies. While partial splenectomy may reduce the risk of infections, total splenectomy remains the predominant choice due to its superior hematologic outcomes (19). Conservative strategies include RBC transfusions, folate supplementation, and recombinant human erythropoietin (rHuEPO) administration to stimulate erythropoiesis, as proposed by Farruggia et al. (20). In the present case, the patient's management emphasized regular CBC monitoring, RBC transfusions when necessary, and ongoing surveillance for jaundice and hepatosplenomegaly. If there is no significant improvement and the timing is deemed appropriate, a splenectomy may be considered. In recent years, substantial advancements have been achieved in the application of gene therapy and genome editing technologies for the treatment of hereditary anemia, particularly in the study of red blood cell membrane disorders. CRISPR-Cas9 technology, as a highly precise gene editing tool, has demonstrated successful application in treating conditions such as β-thalassemia and sickle cell anemia (21). Exagamglogene autotemcel represents the first CRISPR-Cas9-based gene therapy to receive approval from the Food and Drug Administration. This therapeutic approach involves the modification of patients' hematopoietic stem cells to enhance the expression of fetal hemoglobin, thereby alleviating clinical symptoms and reducing the necessity for blood transfusions (22). In addition, the development of targeted viral vector delivery systems has introduced novel prospects for gene therapy. Through design optimization, researchers have improved the efficiency and specificity of these vectors in targeting red blood cell precursor cells. For example, CD90-targeted lentiviral vectors have been shown to effectively deliver gene editing components to hematopoietic stem cells. Although these approaches have not yet been broadly implemented in the clinical treatment of erythrocyte membrane diseases, they offer promising new directions for future research in this area (23). Despite encouraging outcomes in preclinical studies, several challenges remain in the treatment of erythrocyte membrane disorders, including improving targeted delivery efficiency, ensuring long-term expression stability, and mitigating potential immune responses. Therefore, further research is necessary to refine gene editing techniques and delivery systems in order to develop safer and more effective therapeutic strategies.

## Conclusion

We firstly reported a novel frameshift mutation of ANK1 in a neonatal HS case, which expands the variation spectrum of ANK1 in the population. Moreover, this case showed no typical clinical manifestations, and routine examinations failed to provide effective diagnostic evidence. Diagnosis of neonatal or infantile HS remains challenging, necessitating vigilance for atypical presentations such as anemia or previously resolved hyperbilirubinemia. When traditional laboratory findings are negative, genetic testing is necessary for achieving a definitive diagnosis and facilitating targeted therapy.

## Data Availability

The datasets presented in this study can be found in online repositories. The names of the repository/repositories and accession number(s) can be found in the article/Supplementary Material.
